# Hydrogen-rich water increases postharvest quality by enhancing antioxidant capacity in *Hypsizygus marmoreus*

**DOI:** 10.1186/s13568-017-0496-9

**Published:** 2017-12-20

**Authors:** Hui Chen, Jinjing Zhang, Haibo Hao, Zhiyong Feng, Mingjie Chen, Hong Wang, Ming Ye

**Affiliations:** 1 Microbial Resources and Application Laboratory, School of Food Science and Engineering, Hefei University of Technology, 609 Room, No.193, Tunxi Road, Hefei, 230009 China; 2 National Research Center for Edible Fungi Biotechnology and Engineering, Key Laboratory of Applied Mycological Resources and Utilization, Ministry of Agriculture, Shanghai Key Laboratory of Agricultural Genetics and Breeding, Institute of Edible Fungi, Shanghai Academy of Agricultural Sciences, Shanghai, China

**Keywords:** *Hypsizygus marmoreus*, Hydrogen (H_2_), Hydrogen-rich water, Antioxidant activities, Postharvest storage

## Abstract

In the present study, we aimed to assess the effect of hydrogen-rich water (HRW) on the physicochemical characteristics and antioxidant capacity of *Hypsizygus marmoreus* during 12 days of postharvest storage at 4 °C. Different concentrations of HRW (25, 50 and 100%) were tested, and our data showed that 25% HRW treatment had the most significant effect on preservation of nutrients in *H. marmoreus* compared with the control group. In addition, 25% HRW treatment significantly reduced the relative electrolyte leakage rate and malonaldehyde (MDA) content (P < 0.05) and increased anti-superoxide-radical (O_2_^−^) activity compared with the control group. The activities of antioxidants, superoxide dismutase (SOD), catalase (CAT), ascorbate peroxidase (APX) and glutathione reductase (GR) were activated by 25% HRW treatment, and the expression levels of these genes were also induced. These results suggested that HRW treatment could delay rot incidence in mushrooms during storage by regulating antioxidant defense ability. This study supplies a new and simple method to maintain the quality and extend the shelf life of mushrooms.

## Introduction

The edible mushroom *Hypsizygus marmoreus* has become very popular due to a wider appreciation of its long-recognized organoleptic (Harada et al. 2004) and medicinal attributes (Ikekawa 2001; Ohsawa et al. 2007) in East Asia. With the improvements in cultivation technology of *H. marmoreus*, there has recently been a large increase in production and consumer demand. According to data from the Chinese Edible Fungi Association, the daily production of *H. marmoreus* was 167 tons in China in 2012, suggesting a rapidly increasing demand for *H. marmoreus* (Zhang et al. 2016).

Despite its popularity, the fruit bodies of *H. marmoreus* deteriorate rapidly after harvest due to physical damage, microbial attack, water loss, browning, softening and so on, resulting in greatly reduced consumer acceptability (Xing et al. 2007, 2008). Xing et al. (2007, 2008) have reported that they used ^60^Co γ-irradiation and different packaging films to enhance the postharvest quality and improve the shelf life of *H. marmoreus*. While this method increases mushroom shelf life, it also has drawbacks, such as safety considerations, decreased nutritive value, off flavors, high energy consumption, and unsuitability on the industrial scale.

The postharvest senescence of fresh products is often associated with increased oxidative damage to proteins, lipids and nucleic acids by reactive oxygen species (ROS) (Dama et al. 2010; Li et al. 2016; Mittler 2002). ROS were considered toxic by-products of aerobic metabolism, which were disposed of using antioxidants. To protect against ROS damage, mushrooms have developed multiple detoxification systems, including antioxidant enzymatic systems (SOD, POD, CAT, APX) (André et al. 2010).

Recently, some researchers have reported that H_2_ can alleviate various abiotic stresses, including high salinity (Xie et al. 2012; Xu et al. 2013), heavy metal (Cui et al. 2013, 2014) and low temperature (Jin et al. 2013), by regulating the antioxidant defense system. Ren et al. (2016) found that 5% hydrogen-rich water (HRW) treatment significantly decreases the content of ROS, maintains the biomass and polar growth morphology of mycelium, and decreases secondary metabolism under HAc-induced oxidative stress. In kiwifruit, 80% HRW treatment exhibits the most significant effect, displaying reduced rot incidence and better-preserved firmness, and HRW treatment also increases the activities of antioxidants and maintains radical-scavenging activity in kiwifruit (Hu et al. 2014).

In our previous study, we found that HRW treatment significantly improved mycelial growth and biomass by enhancing antioxidant capacity (Zhang et al. 2017). In this study, we aimed to investigate the effect of HRW on the parameters of physical quality and antioxidant capacity of *H. marmoreus* during storage. Our results indicated that HRW treatment delayed the deterioration process, reduced lipid peroxidation, increased antioxidant activity and maintained free-radical-scavenging activity in *H. marmoreus*. These results suggested that HRW treatment was a useful and simple method to maintain the quality and extend the shelf life of mushrooms. To the best of our knowledge, this is the first report on the effect of H_2_ on mushrooms during storage.

## Materials and methods

### Mushroom treatment and storage

In the present study, *H. marmoreus* were collected from Shanghai Bright Esunyes Bio-tech Co., Ltd. in China. Mature mycelia of *H. marmoreus* were scraped with a scraping machine and then added into 15 mL water or 25, 50 and 100% HRW. HRW was kindly supplied by Beijing Hydrovita Beverage Co., Ltd. (Beijing, China), and the H_2_ concentration of fresh HRW was 1.0 mM in a hermetic canister. When the canister was opened, the H_2_ concentration fell to 0.8 mM in 30 min. The H_2_ concentration was maintained at 0.4 mM in 25 °C for at least 12 h. The H_2_ concentration was analyzed by using gas chromatography according to the method of Zhang et al. (2017). After these processes, the mature mycelia were transferred to a growth room for fruiting bodies. The collected mushrooms were transported to the laboratory within 1 h after harvest and then stored in darkness at 4 ± 1 °C and 80% relative humidity (RH) in a crisper.

### Analysis of functional components

Weight loss was determined by weighing the whole mushroom before and after storage. Every ten fruit bodies were considered as one group, and each sample had three groups as three repetitions. Weight loss was expressed as a percentage of the initial weight.

Firmness of the mushroom cap was detected according to (Jiang et al. 2011). A TA.XTplus texture analyzer (Stable Micro Systems Ltd., Co., UK) was used to test cap firmness by using a 2-mm diameter cylindrical probe. During the pretest and penetration, the speed of the probe was kept at 2.0 mm/s. The probe penetrated 5 mm into the samples. Texture Export (version 1.0) from stable micro systems was used to record the force and time data. Firmness was defined as the maximum force according to the force and time curves. The unit of force was N, and each sample was tested ten times.

### Relative conductivity and soluble protein

The electrolyte leakage was essentially determined as described by (Xing et al. 2007). The fruit bodies of *H. marmoreus* (5 g) were cut into four pieces, leaving the pileus intact, and then they were suspended in 40 mL of deionized water in a 100-mL beaker. Electrical conductivity was measured immediately (P0) and again after 10 min (P1). Samples were then boiled for 10 min and cooled down to room temperature, and a final conductivity (P2) was measured. The relative electrolyte leakage rate (RELR) was calculated according to the equation as follows: ${{\left( {{\text{P}}1 - {\text{P}}0} \right)} \mathord{\left/ {\vphantom {{\left( {{\text{P}}1 - {\text{P}}0} \right)} {\left( {{\text{P}}2 - {\text{P}}0} \right)}}} \right. \kern-0pt} {\left( {{\text{P}}2 - {\text{P}}0} \right)}}$ and expressed as a percentage. Each sample was detected for three repetitions.

Soluble protein was detected by a protein kit (Nanjing Jiancheng Bioengineering Institute) using the Coomassie brilliant blue method and monitored at 595 nm. The standard protein concentration used in this assay kit was 0.563 g/L, and the color solution was Coomassie brilliant solution.

### Nutrient component detection

The contents of water, polysaccharide, crude fiber, protein, total sugar and amino acids were detected by the supervision and testing center for edible fungi quality (Shanghai, China), Ministry of Agriculture. Water content detection was performed using a DHG Series Heating and Drying Oven (Shanghai, China); the detection of polysaccharide, crude fiber, protein and total sugar used a Synergy HT microplate reader (Bio-Tek, USA); and the test of amino acid content was performed using an A200 automatic Amino Acid Analyzer (amino, Germany). The test standards for detecting these items were listed in Table 2.

### Malondialdehyde (MDA) and anti-superoxide anion activity assay

During the storage process, the fruit bodies were collected at 0, 3, 6, 9 and 12 days. The fruit bodies were ground into a fine powder in liquid nitrogen, and then the fruit body tissue (1.0 g) was homogenized with 9.0 mL of normal saline. The homogenates were centrifuged at 1000*g* for 10 min at 4 °C. Lipid peroxidation was assayed by estimating the MDA concentration in the supernatant using a commercially available kit (Nanjing Jiancheng Bioengineering Institute, Nanjing, China). The mixture containing mushroom supernatant and reagents was heated at 95 °C for 40 min. The sample was quickly cooled to room temperature and then centrifuged at 4000*g* for 10 min. The absorbance of the supernatant was determined at a wavelength of 532 nm.

The inhibition of superoxide anion (O_2_^−^) activity was performed as described by the inhibition and produced O_2_^−^ Assay Kit (Nanjing Jiancheng Bioengineering Institute, Nanjing, China). In this reaction system, the amount of O_2_^−^ that was inhibited by 1 g of mycelial protein at 37 °C for 40 min was related to the amount of O_2_^−^ inhibited by 1 mg of vitamin C as monitored at 550 nm, and this value was taken as one unit of O_2_^−^ inhibition activity.

### Enzyme activity assay

To test for changes in antioxidant activity, the fruit bodies of *H. marmoreus* were collected at 0, 3, 6, 9 and 12 days during the storage process and immediately ground into a fine powder in liquid nitrogen. Then, the fruit body tissue (1.0 g) was homogenized with 9.0 mL of normal saline, and the sample was centrifuged at 2500*g* for 10 min. The supernatant (50 μL) was used to detect catalase (CAT), superoxide dismutase (SOD), ascorbate peroxidase (APX) and glutathione reductase (GR) activity by using the corresponding assay kits (Nanjing Jiancheng Bioengineering Institute, Nanjing, China). One unit of CAT was defined as the amount of enzyme that decomposed 1 μmol of H_2_O_2_ as monitored at 405 nm. One unit of SOD activity was defined as the amount of oxidative inhibition by SOD up to 50% per gram tissue in 1 mL of reaction solution, and the SOD activity was monitored at 550 nm. One unit of APX was defined as the oxidation of 1 μmol AsA per milligram of protein per minute and was monitored at 290 nm. One unit of GR was defined as 1.0 mg protein, which catalyzes the reduction of 1 nmol NADPH per minute and was monitored at 340 nm.

### Gene expression analysis by qRT-PCR

Approximately 2 μg of total RNA purified from the samples was reverse-transcribed into cDNA by M-MLV reverse transcriptase (Takara) using oligo (dT) as the primer. The expression of four antioxidants at the mRNA level was assessed by quantitative real-time PCR (qRT-PCR), and the 18S ribosomal RNA was selected as the housekeeping gene. Table 1 lists the primer sequences and accession numbers of these genes. The amplification was carried out using the conditions previously described by (Zhang et al. 2015a, b). Three independent biological replicates were performed for each gene, and the relative gene expression was analyzed using the 2^−ΔΔCt^ method described by (Livak and Schmittgen 2001).

**Table 1:** The primers used in qRT-PCR

	Sequences (5′–3′)	Accession number
cat-F	CAACCGTCCGTTTCTCCACT	GBCL00039326
cat-R	CCAATCCCAATTACCTTCCTC	
sod-F	GCAATGGCGGCACTCTGAAA	GBCL00008983
sod-R	CAAGAGCGGGTCCTGGTTCG	
apx-F	TGAACCTCCACCGTACACCA	KYQ43457
apx-R	GGCTTCCGAACCATCCAC	
gr-F	AGCAAGCCTTCCGACGAGCAA	GBCL00010302
gr-R	CGACGGCGATGTAGCCAGCA	
18 s-F	GAGGGACCTGAGAAACG	KC510993
18 s-R	ATAAGACCCGAAAGAGCC	

### Data presentation and statistical analysis

Values are shown as the mean ± SD of three independent experiments with three replicates each. Differences among treatments were analyzed by one-way analysis of variance (ANOVA) combined with Duncan’s multiple range test at a probability of P < 0.05.

## Results

### Effects of HRW on the fruit bodies of *Hypsizygus marmoreus*

**Fig. 1:**
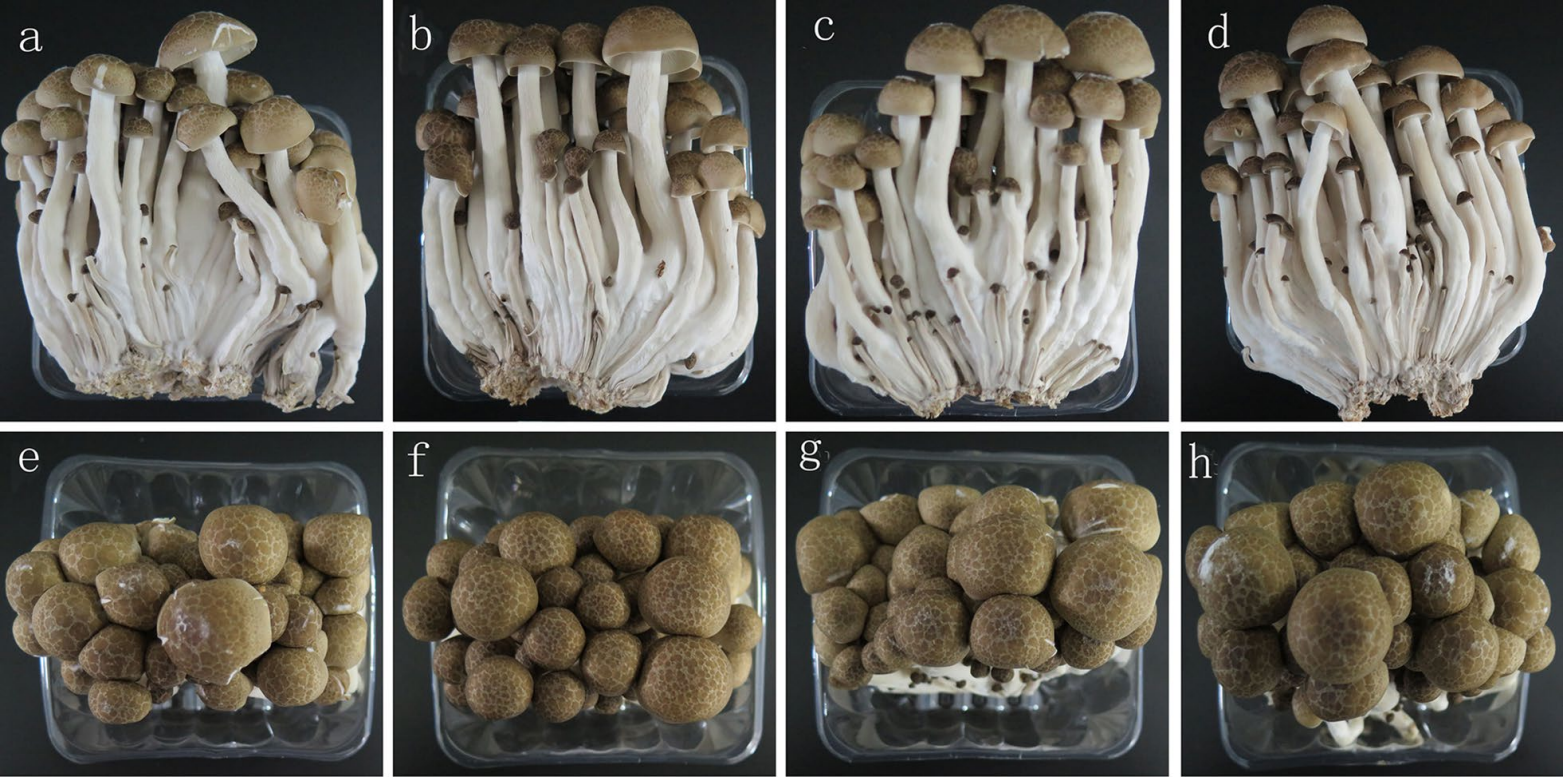
Effects of HRW on sensory quality of *Hypsizygus marmoreus* after 12 days of storage. **a**, **e** Control group; **b**, **f** 25% HRW; **c**, **g** 50% HRW; **d**, **h** 100% HRW

Figure 1 shows the sensory quality of mushrooms with HRW treatment. The mushrooms treated with running water (Con) exhibited a certain degree of cap opening, and there were many aerial mycelia on the cap and stipe in the Con group after 12 days of storage at 4 °C. In contrast, 25% or 50% HRW treatment significantly reduced cap opening as well as the growth of aerial mycelia on the cap and stipe. However, 100% HRW treatment accelerated the rot incidence, and there was a little cap opening. Moreover, reduced growth of aerial mycelia was also observed from 100% HRW treatment. The results suggested that H_2_ was beneficial to the alleviation of wounding.

### Weight loss, firmness, relative electrolyte leakage and soluble proteins

**Fig. 2:**
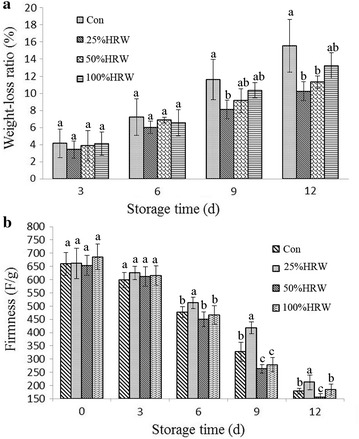
Effects of HRW on weight loss (**a**) and firmness (**b**) in fruit bodies of *Hypsizygus marmoreus* during storage at 4 °C. *Con* control group. The data are presented as the mean ± SD. Bars with different letters denote a statistically significant difference compared with the control according to multiple comparisons (P < 0.05)

There was a strong relationship between these experimental indexes and the edible quality of mushrooms. Figure 2a shows that weight loss was increased as the storage period was extended in both treatments. The weight loss of the three HRW treatment groups was lower than that of the control group. After 9 and 12 days of storage, the HRW treatments had significantly reduced weight loss (P < 0.05), among which 25% HRW was the most efficient treatment for maintaining weight.

As one of the major factors limiting the quality and postharvest shelf life of mushrooms, the firmness of the cap was also tested in the present study. Figure 2b shows the changes in firmness between controls and treated fruit bodies during 12 days of storage. The 25% HRW treatment significantly increased the firmness of mushroom caps compared with the Con group (P < 0.05). However, the 50 and 100% HRW treatments had little effect on the firmness compared with the Con group.

**Fig. 3:**
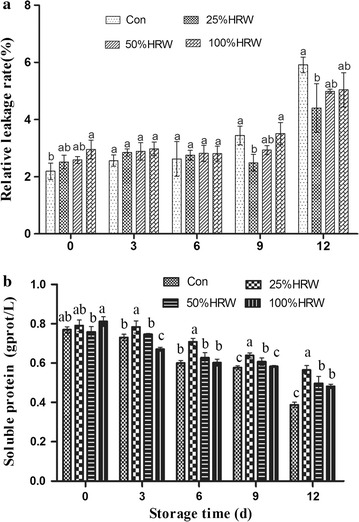
Effects of HRW on RELR (**a**) and soluble protein (**b**) in fruit bodies of *Hypsizygus marmoreus* during storage at 4 °C. *Con* control group. The data are presented as the mean ± SD of three independent experiments. Bars with different letters denote a statistically significant difference compared with the control according to multiple comparisons (P < 0.05)

The RELR slowly increased during the first 6 days of storage and continued to increase throughout storage (Fig. 3a). The RELR in the HRW-treated groups was significantly lower than that of the Con group after 9 and 12 days of storage. In addition, the protein contents in both the HRW and Con treatments were slightly decreased during the 12 days of storage, after which the protein was rapidly decomposed (Fig. 3b). The protein content of the HRW-treated mushrooms was higher than that of the Con group, and the 25% HRW treatment had the most significant effects on maintenance of protein content.

### Nutrient components

The contents of polysaccharide, protein and total sugar were increased at different levels after 25% HRW, 50% HRW and 100% HRW treatments compared with the control group (Table 2). The 50% HRW treatment had the best effects in increasing the contents of polysaccharide, protein and total sugar compared to the 25% HRW and 100% HRW treatments. In addition, the content of eight amino acids, including aspartic acid, serine, glutamic acid, alanine, methionine, tyrosine, phenylalanine and lysine, was increased significantly by HRW treatments compared with the control group, and the fruit bodies treated with 50% HRW had the highest levels of amino acids. Among these amino acids, methionine and phenylalanine are essential amino acids for humans, and glutamic acid and methionine were important flavoring material in the fruit bodies of *H. marmoreus*.

**Table 2:** Effects of HRW on the nutrient components in the fruit body of *Hypsizygus marmoreus*

Test item	Con	25% HRW	50% HRW	100% HRW	Test standard
Water content (%)	89.6 ± 2.31^a^	89.6 ± 1.92^a^	89.7 ± 2.12^a^	89.8 ± 3.28^a^	GB 5009.3-2010
Polysaccharide (%)	0.84 ± 0.032^a^	0.91 ± 0.020^b^	1.13 ± 0.017^c^	0.86 ± 0.030^a^	NY/T 1676-2008
Crude fiber (%)	1.2 ± 0.091^a^	1.1 ± 0.082^a^	1.1 ± 0.075^a^	1.1 ± 0.029^a^	GB/T 5009.10-2003
Protein (%)	2.84 ± 0.152^a^	2.88 ± 0.156^a, b^	2.90 ± 0.201^a, b^	2.87 ± 0.198^a^	GB 5009.5-2010
Total sugar (%)	45.6 ± 3.78^a^	50.8 ± 4.98^b^	52.7 ± 6.09^b^	49.6 ± 2.99^a, b^	GB/T 15672-2009
Aspartic acid (%)	0.128 ± 0.0081^b^	0.115 ± 0.0132^b^	0.138 ± 0.0201^c^	0.095 ± 0.0063^a^	GB/T 5009.124-2003
Threonine (%)	0.100 ± 0.0071^a^	0.096 ± 0.0044^a^	0.098 ± 0.0066^a^	0.098 ± 0.0072^a^	GB/T 5009.124-2003
Serine (%)	0.051 ± 0.0056^b^	0.041 ± 0.0042^b^	0.090 ± 0.0081^c^	0.027 ± 0.0029^a^	GB/T 5009.124-2003
Glutamic acid (%)	0.328 ± 0.0246^a^	0.336 ± 0.0311^a, b^	0.354 ± 0.0382^b^	0.348 ± 0.0471^b^	GB/T 5009.124-2003
Glycine (%)	0.095 ± 0.0071^a^	0.091 ± 0.0091^a^	0.091 ± 0.0055^a^	0.092 ± 0.0060^a^	GB/T 5009.124-2003
Alanine (%)	0.147 ± 0.0186^a^	0.143 ± 0.0092^a^	0.152 ± 0.0118^a^	0.147 ± 0.0181^a^	GB/T 5009.124-2003
Valine (%)	0.101 ± 0.2601^a^	0.098 ± 0.0111^a^	0.100 ± 0.0125^a^	0.099 ± 0.0087^a^	GB/T 5009.124-2003
Methionine (%)	0.094 ± 0.0117^a^	0.117 ± 0.0124^b^	0.123 ± 0.0133^c^	0.109 ± 0.0167^b^	GB/T 5009.124-2003
Isoleucine (%)	0.089 ± 0.0047^a^	0.084 ± 0.0055^a^	0.088 ± 0.0023^a^	0.086 ± 0.0086^a^	GB/T 5009.124-2003
Leucine (%)	0.144 ± 0.0163^a^	0.137 ± 0.0135^a^	0.141 ± 0.0116^a^	0.138 ± 0.0214^a^	GB/T 5009.124-2003
Tyrosine (%)	0.029 ± 0.0013^a^	0.068 ± 0.0034^b^	0.068 ± 0.0055^b^	0.065 ± 0.0074^b^	GB/T 5009.124-2003
Phenylalanine (%)	0.074 ± 0.0037^a^	0.076 ± 0.0024^a, b^	0.080 ± 0.0062^b^	0.079 ± 0.0091^b^	GB/T 5009.124-2003
Lysine (%)	0.027 ± 0.0026^a^	0.049 ± 0.0035^c^	0.036 ± 0.0063^b^	0.028 ± 0.0019^a^	GB/T 5009.124-2003
Histidine (%)	0.059 ± 0.0032^a^	0.058 ± 0.0041^a^	0.058 ± 0.0073^a^	0.065 ± 0.0056^a, b^	GB/T 5009.124-2003
Arginine (%)	0.100 ± 0.0123^a^	0.149 ± 0.0172^b^	0.154 ± 0.0148^b^	0.181 ± 0.0191^c^	GB/T 5009.124-2003
Proline (%)	0.074 ± 0.0043^a^	0.070 ± 0.0064^a^	0.072 ± 0.0025^a^	0.071 ± 0.0043^a^	GB/T 5009.124-2003
Total amino acids (%)	1.64 ± 0.1623^a^	1.69 ± 0.1732^a, b^	1.84 ± 0.1280^b^	1.66 ± 0.0991^a^	GB/T 5009.124-2003

The lowercase letters denote a statistically significant difference compared with the control according to multiple comparisons (P < 0.05)

### MDA and O_2_^−^ contents

**Fig. 4:**
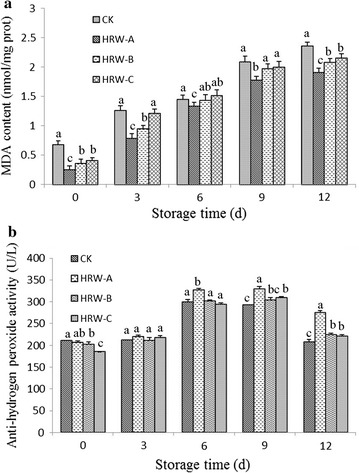
Effects of HRW on the MDA content (**a**) and the inhibition of anti-hydrogen-radical activity (**b**) in fruit bodies of *Hypsizygus marmoreus* during storage at 4 °C. *MDA* malonaldehyde, *Con* control group. The data are presented as the mean ± SD of three independent experiments. Bars with different letters denote a statistically significant difference compared with the control according to multiple comparisons (P < 0.05)

Figure 4a shows that the MDA content rapidly increased during the 9 days of storage and continued to increase throughout storage. Mushrooms treated with HRW had significantly lower MDA contents compared with the Con group (P < 0.05). Among the three HRW treatments, the 25% HRW treatment had the lowest MDA content compared with the Con group. After 12 days of storage, the MDA content of mushrooms treated with 25% HRW was 1.911 mol/mg protein, which was 19.06% lower than that of the Con group.

The anti-hydrogen-peroxide ability was not changed in both control and HRW treatment groups during the first 3 days of storage (Fig. 4b). During the 6–9 days of storage, the anti-hydrogen-peroxide ability was remarkably increased in both the Con and HRW treatment groups (Fig. 4b). After 9 days of storage, the anti-hydrogen-peroxide ability of the 25% HRW group was 292.62 U/L, which was 12.65% higher than that of the Con group. After 12 days of storage, the anti-hydrogen-peroxide ability was reduced, and its lowest level was detected in the Con group (Fig. 4b). The 25% HRW treatment had the highest anti-hydrogen-peroxide ability among the three HRW treatments, and it was 207.87 U/L and 32.42% higher than that of the Con group.

### Antioxidant activities

**Fig. 5:**
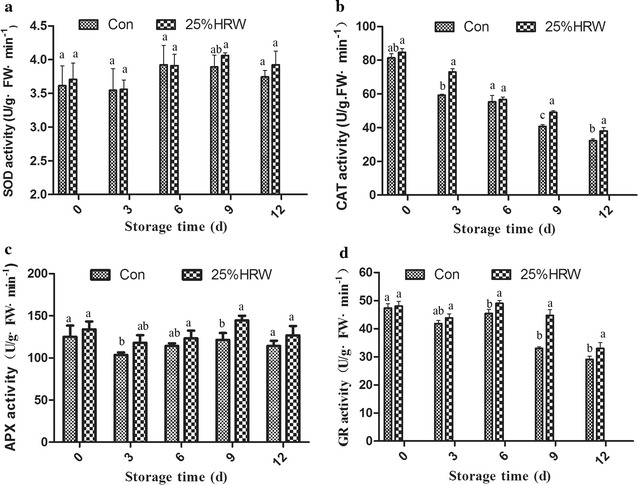
Effects of HRW on the antioxidant enzyme activities of SOD (**a**), CAT (**b**), APX (**c**) and GR (**d**) in fruit bodies of *Hypsizygus marmoreus* during storage at 4 °C. *SOD* superoxide dismutase, *CAT* catalase, *APX* ascorbate peroxidase, *GR* glutathione reductase, *Con* control group. The data are presented as the mean ± SD of three independent experiments. Bars with different letters denote a statistically significant difference compared with the control according to multiple comparisons (P < 0.05)

SOD, CAT, APX and GR are important active free-radical-scavenging enzymes. Figure 5 shows the changes in the activities of these enzymes. The mushrooms treated with 25% HRW showed remarkably higher SOD activity during storage (Fig. 5a). CAT activity in both the Con and HRW treatment groups was decreased during storage, and the highest CAT activity was detected in the 25% HRW treatment (Fig. 5b). APX activity was significantly increased in the 25% HRW treatment group compared with the Con group (P < 0.05) (Fig. 5c). The highest APX activity was detected in mushrooms on the 9th day of storage. GR activity was similar to CAT activity, which decreased during storage (Fig. 5d). The highest GR activity was detected in the 25% HRW treatment group during storage, while its lowest activity was detected on the 12th day of storage.

### Gene expression of antioxidants

**Fig. 6:**
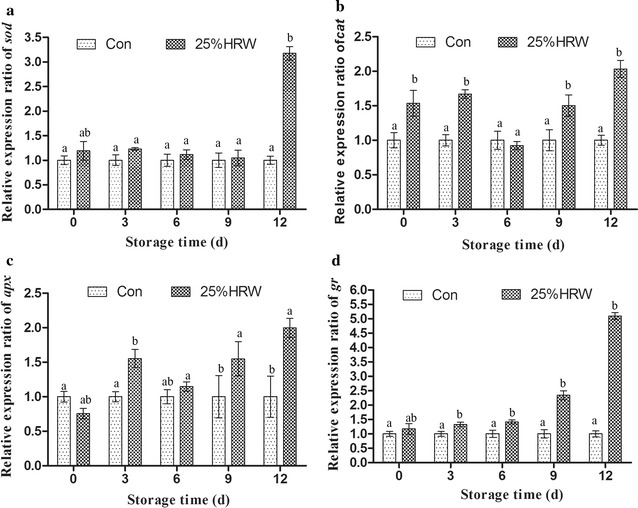
Effects of HRW on antioxidant enzyme gene expression levels of *sod* (**a**), *cat* (**b**), *apx* (**c**) and *gr* (**d**) during storage at 4 °C. *Con* control group. The data are presented as the mean ± SD of three independent experiments

In the present study, we also examined the expressions of *sod*, *cat*, *apx* and *gr* at the mRNA level during storage (Fig. 6). The expression of *cat* was higher in the 25% HRW treatment group compared with the Con group. After 12 days of storage, the expression of *cat* in the HRW treatment group was 2.03-fold that of the Con group (Fig. 6a). The expression of *sod* during 3–9 days of storage was slightly higher than that of the Con group, and its expression of the HRW treatment group after 12 days of storage was 3.18-fold that of the Con group (Fig. 6b). Similar to *cat* expression, the expression of *apx* in the 25% HRW treatment group was higher than that in the Con group, and its expression in the 25% HRW treatment group after 12 days of storage was 2.0-fold that of the Con group (Fig. 6c). The expression of *gr* in the 25% HRW treatment group was 2.34-fold and 5.09-fold that of the Con group after 9 and 12 days of storage, respectively (Fig. 6d).

## Discussion

Mushrooms are highly perishable and tend to lose quality immediately after harvest (Wang et al. 2015). In the present study, we found that treatment with 25% HRW could effectively reduce the rot incidence of *H. marmoreus*, showing reduced growth of aerial mycelia (Fig. 1). Meanwhile, this treatment maintained a lower weight loss (Fig. 2a) and a higher firmness (Fig. 2b) and increased the contents of polysaccharide, protein, total sugar and amino acids (Table 2) in *H. marmoreus* compared with the control group, suggesting that H_2_ was beneficial for delaying senescence in mushrooms.

However, we found that HRW treatment at higher concentrations (75 and 100%) had no effects on inhibiting rot incidence or extending the shelf life of *H. marmoreus*, indicating that HRW did not have dose-dependent effects. In *Medicago sativa*, the maximum inducible response was observed in 10% HRW-pretreated plants (Cui et al. 2013). In Arabidopsis, the maximum inducible response was observed when a 50% saturation of H_2_ was applied alone or followed by NaCl, whereas higher concentrations (75 and 100%) were less effective (Xie et al. 2012). In kiwifruit, treatment with 100% HRW aggravated the rot incidence of fruits (Hu et al. 2014). Therefore, we suggest that the higher level of H_2_ provided by higher concentration HRW (100%) might exceed physiological requirements. In addition, the delay of rot incidence in *H. marmoreus* might be dependent on H_2_ homeostasis.

In kiwifruit, 80% HRW treatment can effectively reduce rot incidence and maintain a higher firmness (Xing et al. 2007), which is similar to our results. In addition to H_2_, the single molecule NO can also improve the quality of *Agaricus bisporus* (Jiang et al. 2011). Further studies have found that H_2_ and NO decrease ROS content and increase antioxidant ability in fruits and mushrooms. In addition, 5% HRW treatment significantly decreases ROS content, maintains the biomass and polar growth morphology of mycelium, and decreases secondary metabolism under HAc-induced oxidative stress in *G. lucidum* (Ren et al. 2016). In plants, H_2_ can also enhance the tolerance of plants to multiple environmental stresses, including drought, cold and salt stresses (Jin et al. 2013). Therefore, HRW might play a role in enhancing antioxidant ability and scavenging ROS.

The production of ROS, such as hydrogen peroxide, is considered as an important effector in mushrooms during storage (Singh and Singh 2013). In our study, we found that HRW treatments could increase anti-hydrogen-peroxide activity, and the 25% HRW treatment led to significant hydrogen peroxide scavenging (Fig. 4b). In kiwifruit, 80% HRW treatment can effectively reduce rot incidence and maintain higher firmness (Hu et al. 2014), leading to increased radical (O_2_^−^ and.OH)—scavenging activity in kiwifruit. Similar with H_2_, NO significantly reduces both O_2_^−^ production rate and H_2_O_2_ content (Xuan et al. 2012; Lai et al. 2011). Ripening in mushrooms and fruits has been described as an oxidative phenomenon, which requires a turnover of ROS, such as H_2_O_2_ and O_2_^−^. However, if excessive ROS were produced, oxidative damage might ensue, making fruits and mushrooms rot quickly. HRW had a positive effect on ROS scavenging and maintaining ROS at a relatively low level.

Meanwhile, H_2_ treatment can induce the activities of antioxidants, such as SOD, CAT and APX, thereby limiting oxidative processes during the storage of fruits (Xu et al. 2013; Hu et al. 2012; Hu et al. 2014). In the present study, we found that 25 and 50% HRW treatments increased the activities of antioxidants, including SOD, CAT, APX and GR (Fig. 5). In addition, we also assessed the gene expression levels of *sod*, *cat*, *apx* and *gr* and found that 25% HRW treatment induced the expression of these genes (Fig. 6), which was consistent with the changes in the activities of these antioxidants. The results were also consistent with those obtained from animals and plants, showing that low doses of H_2_ enhance the activities of antioxidants and the levels of the well-known antioxidant glutathione (GSH), thereby increasing endogenous antioxidant defenses against (Chen et al. 2014; Hong et al. 2010; Jin et al. 2013; Lin et al. 2014; Xu et al. 2013; Wang et al. 2016). These results indicated that HRW treatment could help control ROS content by activating the antioxidant defense system, thereby alleviating oxidative damage and delaying senescence.

Moreover, ROS accumulation in the cell damages membrane structure and function, triggering membrane lipid peroxidation and inducing membrane breakdown (Juan et al. 2011; Oz et al. 2015). In this study, MDA content (Fig. 4a) and RELR (Fig. 3a) increased during storage, which was consistent with the increased ROS level (Fig. 4b). HRW treatment significantly reduced these increases in MDA and RELR. During the process of fruit storage, lipoxygenase activity, conjugated diene and MDA contents are positively correlated with ROS levels, indicating that the extent of membrane lipid peroxidation depends on the ROS level (Chomkitichai et al. 2014). These results suggested that HRW treatment could reduce the rot incidence in mushrooms by decreasing membrane permeability and lipid peroxidation.

In conclusion, our study showed that pretreatment with 25% HRW could effectively inhibit rot incidence and extend the shelf life of *H. marmoreus*. Moreover, 25% HRW treatment maintained higher firmness and delay weight loss. In addition, 25% HRW treatment reduced lipid peroxidation by decreasing the MDA content and increasing anti-hydrogen-radical activity. Furthermore, 25% HRW treatment also increased the activities of antioxidants, including SOD, CAT, APX and GR. These results suggested that HRW treatment had a potential role in the preservation of *H. marmoreus* by enhancing the antioxidant ability.

## Abbreviations

HRW: hydrogen-rich water
MDA: malonaldehyde
O_2_^−^: superoxide radical
CAT: catalase
SOD: superoxide dismutase
APX: ascorbate peroxidase
GR: glutathione reductase
ROS: reactive oxygen species

## References

[CR1] André CM, Larondelle Y, Evers D (2010). Dietary antioxidants and oxidative stress from a human and plant perspective: a review. Curr Nutr Food Sci.

[CR3] Chen M, Cui WT, Zhu KK, Xie YJ, Zhang CH, Shen WB (2014). Hydrogen-rich water alleviates aluminum-induced inhibition of root elongation in alfalfa via decreasing nitric oxide production. J Hazard Mater.

[CR4] Chomkitichai W, Chumyam A, Rachtanapun P, Uthaibutra J, Saengnil K (2014). Reduction of reactive oxygen species production and membrane damage during storage of ‘Daw’ longan fruit by chlorine dioxide. Sci Hortic.

[CR5] Cui WT, Gao CY, Fang P, Lin GQ, Shen WB (2013). Alleviation of cadmium toxicity in *Medicago sativa* by hydrogen-rich water. J Hazard Mater.

[CR6] Cui WT, Fang P, Zhu KK, Mao Y, Gao C, Xie YJ, Wang J, Shen WB (2014). Hydrogen-rich water confers plant tolerance to mercury toxicity in alfalfa seedlings. Ecotoxicol Environ Saf.

[CR7] Dama CL, Kumar S, Mishra BK, Shukla KB, Mathur S, Doshi A (2010). Antioxidative enzymatic profile of mushrooms stored at low temperature. J Food Sci Technol.

[CR10] Harada A, Gisusi S, Yoneyama S, Aoyama M (2004). Effects of strain and cultivation medium on the chemical composition of the taste components in fruit-body of *Hypsizygus marmoreus*. Food Chem.

[CR11] Hong Y, Chen S, Zhang JM (2010). Hydrogen as a selective antioxidant: a review of clinical and experimental studies. J Int Med Res.

[CR12] Hu LY, Hu SL, Wu J, Li YH, Zheng JL, Wei ZJ, Liu J, Wang HL, Liu YS, Zhang H (2012). Hydrogen sulfide prolongs postharvest shelf life of strawberry and plays an antioxidative role in fruits. J Agric Food Chem.

[CR13] Hu HL, Li PX, Wang YN, Gu RX (2014). Hydrogen-rich water delays postharvest ripening and senescence of kiwifruit. Food Chem.

[CR15] Ikekawa T (2001). Beneficial effects of edible and medicinal mushrooms on health care. Int J Med Mushrooms.

[CR16] Jiang TJ, Zheng XL, Li JR, Jing GX, Cai LY, Ying TJ (2011). Integrated application of nitric oxide and modified atmosphere packaging to improve quality retention of button mushroom (*Agaricus bisporus*). Food Chem.

[CR17] Jin QJ, Zhu KK, Cui WT, Xie YJ, Han B, Shen WB (2013). Hydrogen gas acts as a novel bioactive molecule in enhancing plant tolerance to paraquatinduced oxidative stress via the modulation of heme oxygenase-1 signalling system. Plant Cell Environ.

[CR18] Juan K, Wang HM, Jin CH (2011). Changes of reactive oxygen species and related enzymes in mitochondrial respiration during storage of harvested peach fruits. Agric Sci China.

[CR19] Lai TF, Wang YY, Li BQ, Qin G, Tian S (2011). Defense responses of tomato fruit to exogenous nitric oxide during postharvest storage. Postharvest Biol Technol.

[CR21] Li D, Qin X, Tian P, Wang J (2016). Toughening and its association with the postharvest quality of king oyster mushroom (*Pleurotus eryngii*) stored at low temperature. Food Chem.

[CR01] Lin YT, Zhang W, Qi F, Cui WT, Xie YJ, Shen WB (2014) Hydrogen-rich water regulates cucumber adventitious root development in a heme oxygenase-1/carbon monoxide-dependent manner. J Plant Physiol 171:1–8 10.1016/j.jplph.2013.08.009 24331413

[CR22] Livak KJ, Schmittgen TD (2001). Analysis of relative gene expression data using real-timequantitative PCR and the 2^−∆∆CT^ method. Methods.

[CR23] Mittler R (2002). Oxidative stress, antioxidants and stress tolerance. Trends Plant Sci.

[CR25] Ohsawa I, Ishikawa M, Takahashi K, Watanabe M, Nishimaki K, Yamagata K, Katsura K, Katayama Y, Asoh S, Ohta S (2007). Hydrogen acts as a therapeutic antioxidant by selectively reducing cytotoxic oxygen radicals. Nat Med.

[CR26] Oz AT, Ulukanli Z, Bozok F, Baktemur G (2015). The postharvest quality, sensory and shelf life of *Agaricus bisporus* in active map. J Food Process Preserv.

[CR28] Ren A, Liu R, Miao ZG, Zhang X, Cao PF, Chen TX, Li CY, Shi L, Jiang AL, Zhao MW (2016). Hydrogen-rich water regulates effects of ROS balance on morphology, growth and secondary metabolism via glutathione peroxidase in *Ganoderma lucidum*. Environ Microbiol.

[CR29] Singh SP, Singh Z (2013). Controlled and modified atmospheres influence chilling injury, fruit quality and antioxidative system of Japanese plums (*Prunus salicina* Lindell). Int J Food Sci Technol.

[CR31] Wang ZG, Chen LJ, Yang H, Wang AJ (2015). Effect of exogenous glycine betaine on qualities of button mushrooms (*Agaricus bisporus*) during postharvest storage. Eur Food Res Technol.

[CR32] Wang Y, Duan XL, Xu S, Wang R, Ouyang ZZ, Shen WB (2016). Linking hydrogen-mediated boron toxicity tolerance with the improvement of root elongation, water status and redox balance: a case study for rice. Ann Bot.

[CR33] Xie YJ, Mao Y, Lai DW, Zhang W, Shen WB (2012). H_2_ enhances *Arabidopsis* salt tolerance by manipulating ZAT10/12-mediated antioxidant defence and controlling sodium exclusion. PLoS ONE.

[CR34] Xing ZT, Wang YS, Feng ZY, Zhao ZH, Xing ZT (2007). Effect of ^60^Co-irradiation on postharvest quality and selected enzyme activities of *Hypsizygus marmoreus* fruit bodies. J Agric Food Chem.

[CR35] Xing ZT, Wang YS, Feng ZY, Tan Q (2008). Effect of different packaging films on postharvest quality and selected enzyme activities of *Hypsizygus marmoreus* mushrooms. J Agric Food Chem.

[CR36] Xu S, Zhu SS, Jiang YL, Wang N, Wang R, Shen WB (2013). Hydrogen rich water alleviates salt stress in rice during seed germination. Plant Soil.

[CR02] Xuan W, Xu S, Li MY, Han B, Zhang B, Zhang J, Lin YT, Huang JJ, Shen WB, Cui J (2012) Nitric oxide is involved in hemin-induced cucumber adventitious rooting process. J Plant Physiol 169:1032-1039 10.1016/j.jplph.2012.02.021 22579358

[CR37] Zhang XN, Zhao XQ, Wang ZQ, Shen WB, Xu XM (2015). Protective effects of hydrogen-rich water on the photosynthetic apparatus of maize seedlings (*Zea mays* L.) as a result of an increase in antioxidant enzyme activities under high light stress. Plant Growth Regul.

[CR38] Zhang JJ, Ren A, Chen H, Zhao MW, Shi L, Chen MJ, Wang H, Feng ZY (2015). Transcriptome analysis and its application in identifying genes associated with fruiting body development in basidiomycete *Hypsizygus marmoreus*. PLoS ONE.

[CR39] Zhang JJ, Chen H, Chen MJ, Wang H, Song XX, Feng ZY (2016). Construction and application of a gene silencing system using a dual promoter silencing vector in *Hypsizygus marmoreus*. J Basic Microbiol.

[CR40] Zhang JJ, Hao HB, Chen MJ, Wang H, Feng ZY, Chen H (2017). Hydrogen-rich water alleviates the toxicities of different stresses to mycelial growth in *Hypsizygus marmoreus*. AMB Express.

